# Survey of Torus Palatinus in Patients with End-Stage Renal Disease Undergoing Hemodialysis

**DOI:** 10.1155/2018/1356910

**Published:** 2018-12-06

**Authors:** Shao-Yu Tai, Chia-Lin Hsu, Aileen I. Tsai, Chih-Chun Chang, I-Kuan Wang, Cheng-Hao Weng, Wen-Hung Huang, Ching-Wei Hsu, Pei-Ching Chang, Tzung-Hai Yen

**Affiliations:** ^1^Department of Pediatric Dentistry, Chang Gung Memorial Hospital, Linkou, Taiwan; ^2^Department of Clinical Pathology, Far Eastern Memorial Hospital, New Taipei, Taiwan; ^3^Department of Nephrology, China Medical University Hospital and College of Medicine, China Medical University, Taichung, Taiwan; ^4^Department of Nephrology and Clinical Poison Center, Chang Gung Memorial Hospital and College of Medicine, Chang Gung University, Linkou, Taiwan; ^5^Kidney Research Center, Chang Gung Memorial Hospital, Linkou, Taiwan; ^6^Center for Tissue Engineering, Chang Gung Memorial Hospital, Linkou, Taiwan

## Abstract

**Introduction:**

This study attempted to survey the oral findings of hemodialysis patients and analyze the prevalence and predictors for torus palatinus** (**TP) in this patient population.

**Methods:**

A total of 322 hemodialysis patients were recruited. Patients were organized into two groups, based on the presence (n=93) or absence (n=229) of TP. Demographic, laboratory, and dialysis-related data were obtained for analysis.

**Results:**

The prevalence of TP was 28.9% in this study. Patients with TP were younger in age [57.8±10.0 (37.4-86.9) versus 62.4±12.3 (25.0-87.8) years old;* P*=0.001] and predominantly female (60.2% versus 38.0%;* P*<0.001), compared to patients without TP. All TPs (100.0%) were symmetrical and located along the midpalatal suture. Most TPs were flat-shaped (55.9%) and near premolars (78.5%). The blood tests revealed higher blood concentrations of phosphate (5.4±1.1 versus 4.9±1.1 mg/dL;* P*=0.001) and lower blood concentrations of bicarbonate (20.9±2.4 versus 22.0±2.3 mmol/L;* P*<0.001) in patients with TP. Multivariate regression modeling showed that younger age [odds ratio (OR) 0.968; 95% confidence interval (CI) 0.939–0.982;* P*<0.001], female gender (OR 2.305; 95% CI 1.374–3.867;* P*=0.002), higher blood concentration of phosphate (OR 1.411; 95% CI 1.110–1.794;* P*=0.005), and lower blood concentration of bicarbonate (OR 0.868; 95% CI 0.791–0.994;* P*=0.040) were significant predictors for TP.

**Conclusion:**

The prevalence of TP is 28.9%, and the majority of patients suffering TP are female. Younger age, female gender, elevated blood concentration of phosphate, and lower blood concentration of bicarbonate are predictors for TP.

## 1. Introduction

Oral tori are usually found accidentally during dental examinations. Torus palatinus (TP) develops along the midpalatal suture, while torus mandibularis occurs on the lingual surface of the mandible. Although torus may start to form in early adult life, it may not become visible until the middle age of the adulthood. [1] Histologically speaking, oral tori are hyperplastic bones comprising cortical and trabecular bones. Since oral tori are benign lesions, surgical removal usually will not be necessary, unless the lesions are large enough to interfere with speech, mastication, and dental prosthesis or lead to ulceration [2].

The etiology of oral torus is complex and may involve environmental factors such as occlusal (biting) forces as well as genetic factors which modify the risk for developing torus. The fact that oral tori are always found in adulthood suggests not only a genetic but also environmental and functional origins. [3, 4] Hence, the etiology of torus could be multifactorial, including, for example, genetics, environment, age, sex, etc.

Torus formations could also be the outcome of biomechanical forces [5] which decrease cortical and increase trabecular bone masses. In a cross-sectional study, Padbury et al. [5] found that subjects with primary hyperparathyroidism were prone to oral tori and reductions in radicular lamina dura. In another study, Rai et al. [6] found that loss of lamina dura, ground-glass appearance, and reduction in mandibular cortical width were common in subjects with primary hyperparathyroidism, and these findings correlated with increased parathyroid hormone concentrations in blood. In addition, after reviewing the oral findings associated with primary, secondary, and tertiary hyperparathyroidism, Palla et al. [7] reported that bony pathologies mostly occurred in mandible (40.8%), followed by maxilla (29.4%). Pathologies occurring in both jaw bones were found in 29.8% of the cases reviewed.

Most clinical investigations on the etiology of oral tori have focused on genetic and environmental factors, and few have investigated factors related to bone and mineral metabolism. Based on a study conducted in 2012, Sismans et al. [8] reported a prevalence rate of TP (41.7%) in patients undergoing peritoneal dialysis (PD) which was higher than the prevalence reported in the TP population without dialysis (4.1%). [8, 9] They also found that patients with a TP size > 2 cm underwent longer PD treatment compared to patients with a TP size < 2 cm (*P* = 0.009). The authors went on to propose that the higher prevalence of TP in PD patients and the association between PD duration and TP size might be attributable to renal osteodystrophy. The prevalence rates of TP in hemodialysis (23.5%) and peritoneal dialysis patients (34.3%) have also been reported by Chao et al. [10] and Hsu et al. [2], respectively. In both studies, the blood concentrations of the intact parathyroid hormone in patients did not differ significantly between those with and without oral tori (*P* = 0.611 and* P* = 0.126, respectively). Furthermore, no significant differences were seen between patients with oral tori and those without in inflammatory variables such as log high-sensitivity C-reactive protein (*P* = 1.000 and* P* = 0.147, respectively) and nutritional variables such as albumin (*P* = 0.247 and* P* = 0.790, respectively). [2, 10] Hence, it appears that neither hyperparathyroidism nor inflammation-malnutrition syndrome contributes to the risk of developing torus in dialysis patients.

Therefore, the objective of this study was to examine the oral findings of a larger group of hemodialysis patients to determine the prevalence and predictors for TP in this patient population.

## 2. Materials and Methods

### 2.1. Ethical Statement

This study was completed in accordance with the Declaration of Helsinki and was approved by the Medical Ethics Committee of the Chang Gung Memorial Hospital. The Institutional Review Board numbers assigned to the study were 102-2761B and 104-6913C.

### 2.2. Patients

This prospective observational study included 322 hemodialysis patients recruited from the Taoyuan branch of the Chang Gung Memorial Hospital, Taiwan, between August 2016 and December 2016. The patients aged 61.1 ± 11.8 (25.0-87.8) years. Patients were stratified into 2 groups based on the presence or absence of TP.

### 2.3. Diagnosis of TP

**Figure 1:**
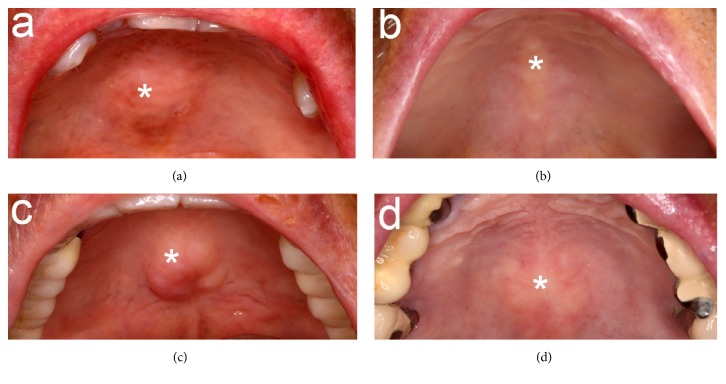
Torus palatinus. The torus palatinus (marked with asterisks) is an exophytic bony mass forming along the midpalatal suture. Intraoral views of four hemodialysis patients present the following types of torus palatinus: flat (a), spindle (b), nodular (c), and lobular (d).

Investigations of the oral cavity consist of inspection and palpation. The size of TP was determined based on the maximum elevation of the outgrowth and was classified as ≥ 2 cm or < 2 cm using a periodontal probe. [11] The shapes of TP were categorized as flat, spindle, nodular, or lobular [12] (Figure 1). The locations of TP were near incisors, premolars, or molars.

### 2.4. Molar Relationship and Oral Hygiene

The molar relationships were categorized as none, class I, II, or III, according to Angle classification. [13] The oral hygiene was recorded using the Turesky-Gilmore-Glickman plaque index (a modification of the Quigley-Hein plaque index). [14]

### 2.5. Statistical Analysis

Student's t-test was used for parametric variables and Chi-Square or Fisher's exact test, for nonparametric variables. Univariate binary logistic regression analysis was performed to analyze the predictors for TP. To control for confounding factors, multivariate binary logistic regression analysis was performed to analyze the significant predictors after univariate analysis. A* P* value less than 0.05 was chosen as the significance threshold to reject the null hypothesis. All analyses were performed using IBM SPSS Statistics Version 20.0.

## 3. Results

### 3.1. Subject Characteristics

The prevalence rate of TP in this study was 28.9% (Table 1). Patients with TP were younger in age than those without TP [57.8±10.0 (37.4-86.9) versus 62.4±12.3 (25.0-87.8) years old;* P*=0.001], and there were more female patients with TP than their male counterparts (60.2% versus 38.0%;* P *< 0.001). There were no significant differences in other baseline variables between the two groups (*P* > 0.05).

### 3.2. Laboratory Findings

Blood tests revealed that patients with TP had higher blood concentrations of phosphate (5.4 ± 1.1 versus 4.9 ± 1.1 mg/dL,* P* = 0.001) and lower blood concentrations of bicarbonate (20.9 ± 2.4 versus 22.0 ± 2.3 mmol/L,* P *< 0.001) than patients without TP (Table 1). Although patients with TP had lower blood concentrations of the intact parathyroid hormone than patients without TP, the difference was not significant (314.1 ± 298.1 versus 330.4 ± 317.1 pg/mL,* P* = 0.671). Meanwhile, no significant differences in inflammatory variables such as high-sensitivity C-reactive protein (6.5 ± 10.2 versus 7.1 ± 11.4 mg/L;* P* = 0.676) and nutritional variables such as albumin (4.0 ± 0.3 versus 3.9 ± 0.3 g/dL;* P* = 0.108) were seen between patients with TP and those without.

### 3.3. Dialysis-Related Data

Table 1 also shows that there are no significant differences in dialysis-related data between the two groups, such as urea reduction ratio, Kt/V, time-averaged concentration of urea, and normalized protein catabolic rate (*P* > 0.05).

### 3.4. Clinical Findings of TPs

Oral examinations revealed that all TPs (100.0%) were symmetrical and located along the midpalatal suture (Table 2). The majority of TPs were near premolars (78.5%). Most were flat-shaped (55.9%) or spindle-shaped (22.6%). Approximately half of the TPs were 2cm or larger (49.5%), and the remaining half (50.5%), smaller than 2cm.

### 3.5. Molar Relationship and Plaque Index

The molar relationship could not be identified in approximately half of the cases (55.0%) due to the loss of first molars (Table 3). For the rest, there was no significant difference in molar relationships between the two groups studied (*P* = 0.198). Table 3 also shows that most hemodialysis patients suffer poor oral hygiene, and three-fourths of the patients have plaque index scores of 3 or 4. However, there is no significant difference in the plaque index between the two groups (*P* = 0.336).

### 3.6. Predictors for TP

The regression analysis results are presented in Table 4. Multivariate regression modeling showed that younger age (OR 0.968; 95% CI 0.939–0.982;* P* < 0.001), female gender (OR 2.305; 95% CI 1.374–3.867;* P *= 0.002), higher blood concentration of phosphate (OR 1.411; 95% CI 1.110–1.794;* P *= 0.005), and lower blood concentration of bicarbonate (OR 0.868; 95% CI 0.791–0.994;* P *= 0.040) were significant predictors for TP.

## 4. Discussion

The literature on the prevalence of TP in hemodialysis patients has been limited. Not only did the current research study a larger patient population (n = 322), but it was also the first to report younger age, female gender, increased blood concentration of phosphate, and decreased blood concentration of bicarbonate as predictors for TP.

Uremia-related changes in facial bone structures have been reported in literature [15–17]. Bakathir et al. [15] described the progressive enlargement of facial bones of a 21-year-old female uremic patient whose facial enlargement involved the maxilla and caused facial and dental deformities. Lopes et al. [16] presented two female uremic patients with facial disfigurement affecting the maxilla and the mandible. Raubenheimer et al. [17] also reported two female uremic cases with extensive jaw lesions due to secondary hyperparathyroidism. In the current study, higher blood concentrations of phosphate (*P* = 0.001) and lower blood concentrations of bicarbonate (*P* < 0.001) were found in patients with TP than those without, and both the blood concentrations of phosphate (*P* = 0.032) and bicarbonate (*P* = 0.019) were deemed significant predictors for TP after further analysis. Although patients with TP had lower blood concentrations of the intact parathyroid hormone than patients without TP, the difference was not significant (*P* = 0.671). As reported by previous investigations [18, 19], hyperphosphatemia, elevated blood concentration of fibroblast growth factor 23, reduction in active vitamin D synthesis, and tendency toward hypocalcemia are all potent stimuli for secondary hyperparathyroidism. Metabolic acidosis is another strong stimulus for secondary hyperparathyroidism, [20] and the blood concentration of the intact parathyroid hormone has been shown to be higher in hemodialysis patients with sodium bicarbonate supplementation than in those without supplementation therapy (*P* < .001).[21] Further studies will be required to delineate the complex interactions between the risk of developing TP and hyperparathyroidism in hemodialysis patients.

According to Hsu et al., [2] TP develops in 4.1% to 60.5% of the general population. However, studies have reported different prevalence rates of TP in different ethnic groups. For example, Chiang et al. [22] reported a prevalence rate of 21.1% by studying a group of 2050 patients in Taiwan. For the population groups such as hemodialysis patients, the prevalence of TP as found in the current study was 28.9%, while previous studies have reported prevalence rates for oral tori of 23.5% in hemodialysis patients, [10] and 41.6% [8] or 34.3% [2] in peritoneal dialysis patients.

In the current study, there were more females in the patient group with TP than in the group without TP (60.2% versus 38.0%,* P* < 0.001). Female gender was also found to be a significant predictor for TP (*P* = 0.001) after further analysis. According to Gorsky et al., [23] idiopathic TP is vertically transmitted as an autosomal dominant trait linked to X chromosome. Females were prone to developing TP, with the prevalence rates ranging from 5.7% to 70.5%, [8–12, 22, 24–33] although one research team reported a higher prevalence rate of TP in males than females. [34] Previous studies [2, 10] conducted by the same research team for the present study also found TP to be more common in females than males. However, no significant difference in the prevalence of torus was found between the two genders in a study of twins. [35]

Patients with TP were younger in age than those without TP in this study (57.8 ± 10.0 versus 62.4 ± 12.3 years old;* P* = 0.001), and young age was found to be a significant predictor for TP (*P* < 0.001) after further analysis. Previously, researchers have reported that most tori develop between the third and fourth decade of life. [25, 26, 28, 36] Increased occurrence of oral tori has been found to correlate with age. [22] The average age of ESRD patients participating in the present study was 61.1 years, much older than participants in previous studies recruited from schools [12, 29, 32], dental clinics [11, 22, 25, 30, 33, 36, 37], or general populations [9, 34, 38] where ages distributed more evenly. Therefore, the finding in the current study that hemodialysis patients with TP were younger than those without TP might not have come as a total surprise.

Flat-shaped TP was the most common type in the current study (55.9%), whereas Reichart et al., [25] Sismans et al., [8] and Jainkittivong et al. [12] have reported that spindle-shaped TP was more common. The finding in the current study mirrors those from earlier studies [2, 10] conducted by the same research team for the current study as well as other investigations [8, 12, 25, 34] where flat-shaped TP was found to be more common than the spindle-shaped type. Additionally, all tori palatinus in this study were symmetrical and located along the midpalatal suture. However, most tori palatinus have been found near premolars (78.5%). Hiremath et al. [32] and Hsu et al. [2] reported that TP was often located in the combined premolar-molar area. Gorsky et al. [11] further reported that the prevalence of TP in the combined premolar-molar area increased with age. Meanwhile, patients with TP ≥ 2 cm in this study underwent longer hemodialysis treatment than patients with TP < 2cm, although the difference was not statistically significant (116.2 ± 99.8, versus 91.2 ± 66.8 months;* P* = 0.159). This finding echoes the observation made by Sismans et al. [8] that patients with a TP > 2 cm underwent longer PD treatment than those with a TP < 2 cm (6.8 ± 3.6 versus 3.5 ± 2.6 years;* P* = 0.009).

In summary, the prevalence of TP in this study was 28.9%, and the majority of patients with TP were female (60.2%). This is the first report to show that younger age, female gender, elevated blood concentration of phosphate, and decreased blood concentration of bicarbonate are predictors for TP in hemodialysis patients. Further studies will be warranted to further validate these findings. The limitations of this study included small sample size and lack of torus mandibularis analysis.

## Figures and Tables

**Table 1:** Baseline characteristics, laboratory findings, and dialysis-related data of hemodialysis patients with and without TP (n = 322).

Variable	All patients (n = 322)	Patients with TP (n = 93)	Patients without TP (n = 229)	*P* value
Baseline characteristics				
Age, year	61.1 ± 11.8	57.8 ± 10.0	62.4 ± 12.3	0.001*∗∗*
Female gender, n (%)	143 (44.4)	56 (60.2)	87 (38.0)	<0.001*∗∗∗*
Hypertension, n (%)	164 (50.9)	48 (51.6)	116 (50.7)	0.876
Diabetes mellitus, n (%)	114 (35.4)	32 (34.4)	82 (35.8)	0.812
Dialysis duration, months	107.1 ± 82.3	103.6 ± 85.2	108.6 ± 81.3	0.622
Alcohol consumption, n (%)	11 (3.4)	4 (4.3)	7 (3.1)	0.577
Betel nut chewing, n (%)	12 (3.7)	5 (5.4)	7 (3.1)	0.319
Cigarette habit, n (%)	28 (8.6)	7 (7.5)	21 (9.2)	0.635
Bruxism habit, n (%)	33 (10.2)	11 (11.8)	22 (9.6)	0.551

Laboratory findings				
Blood urea nitrogen, mg/dL	67.4 ± 17.7	68.9 ± 15.3	66.8 ± 18.6	0.341
Creatinine, mg/dL	10.3 ± 2.1	10.4 ± 1.9	10.3 ± 2.1	0.566
Estimated glomerular filtration rate, mL/min/1.73 m^2^	4.8 ± 1.1	4.5 ± 1.1	4.8 ± 1.1	0.017*∗*
Uric acid, mg/dL	6.7 ± 1.3	7.0 ± 1.2	6.7 ± 1.3	0.036*∗*
Sodium, mEq/L	137.8 ± 2.8	137.7 ± 2.8	137.9 ± 2.8	0.716
Potassium, mEq/L	4.7 ± 0.7	4.8 ± 0.6	4.6 ± 0.7	0.013*∗*
Chloride, mEq/L	99.1 ± 2.9	99.1 ± 3.1	99.1 ± 2.8	0.925
Calcium, mg/dL	9.7 ± 0.9	9.6 ± 0.9	9.7 ± 1.0	0.771
Phosphate, mg/dL	5.0 ± 1.2	5.4 ± 1.1	4.9 ± 1.1	0.001*∗∗*
Bicarbonate, mmol/L	21.7 ± 2.4	20.9 ± 2.4	22.0 ± 2.3	<0.001*∗∗∗*
Fasting glucose, mg/dL	117.0 ± 53.0	114.5 ± 54.2	118.0 ± 52.6	0.596
Albumin, g/dL	4.0 ± 0.3	4.0 ± 0.3	3.9 ± 0.3	0.108
Alkaline phosphatase, U/L	75.0 ± 41.3	73.5 ± 44.1	75.6 ± 40.2	0.684
Total cholesterol, mg/dL	160.6 ± 33.2	168.2 ± 33.3	157.5 ± 32.8	0.009*∗∗*
High-density lipoprotein, mg/dL	43.2 ± 14.2	42.8 ± 14.0	43.3 ± 14.3	0.750
Low-density lipoprotein, mg/dL	106.2 ± 72.6	110.6 ± 68.1	104.4 ± 74.4	0.492
Triglyceride, mg/dL	146.5 ± 117.0	148.8 ± 105.3	145.6 ± 121.6	0.826
Aspartate aminotransferase, U/L	22.1 ± 8.9	22.3 ± 7.9	22.0 ± 9.2	0.813
Alanine aminotransferase, U/L	17.5 ± 12.9	18.8 ± 13.4	16.9 ± 12.8	0.220
Gamma-glutamyl transferase, U/L	32.2 ± 42.9	32.4 ± 45.7	32.1 ± 41.9	0.955
White blood cell count, 10^3^/uL	6.4 ± 1.9	6.8 ± 2.1	6.2 ± 1.8	0.014
Red blood cell count, 10^6^/uL	3.6 ± 0.5	3.6 ± 0.5	3.6 ± 0.6	0.339
Hemoglobin, g/dL	10.4 ± 1.2	10.3 ± 1.2	10.5 ± 1.2	0.316
Hematocrit, %	32.0 ± 3.6	31.7 ± 3.5	32.1 ± 3.6	0.335
Mean corpuscular volume, fL	89.0 ± 7.5	89.1 ± 6.5	89.0 ± 7.9	0.903
Mean corpuscular hemoglobin, pg/Cell	29.0 ± 2.8	29.0 ± 2.5	28.9 ± 3.0	0.928
Mean corpuscular hemoglobin concentration, gHb/dL	32.5 ± 1.0	32.5 ± 0.9	32.5 ± 1.1	0.753
Red blood cell distribution width, %	14.5 ± 1.4	14.4 ± 1.5	14.6 ± 1.3	0.135
Platelet count, 10^3^/uL	184.3 ± 63.7	200.3 ± 61.8	177.8 ± 63.4	0.004*∗∗*
Iron, ug/dL	70.2 ± 53.6	76.1 ± 89.6	67.8 ± 28.1	0.208
Total iron binding capacity, ug/dL	256.9 ± 49.8	262.7 ± 50.2	254.6 ± 49.5	0.186
Ferritin, ng/mL	297.7 ± 300.4	285.4 ± 242.4	303.0 ± 321.3	0.634
Intact parathyroid hormone, pg/mL	325.7 ± 311.4	314.1 ± 298.1	330.4 ± 317.1	0.671
High sensitivity C-reactive protein, mg/L	6.9 ± 11.1	6.5 ± 10.2	7.1 ± 11.4	0.676

Dialysis related data				
Residual glomerular filtration rate, mL/min	3.5 ± 3.4	3.5 ± 3.4	3.5 ± 3.4	0.893
Urea reduction ratio	0.8 ± 0.1	0.8 ± 0.1	0.8 ± 0.1	0.569
Kt/V	1.8 ± 0.4	1.8 ± 0.3	1.8 ± 0.4	0.666
Time-averaged concentration of urea, mg/dL	39.6 ± 9.8	40.5 ± 8.9	39.2 ± 10.1	0.288
Normalized protein catabolic rate, g/kg/day	1.2 ± 0.4	1.2 ± 0.4	1.2 ± 0.4	0.617

TP: torus palatinus; *∗P* < .05, *∗∗P* < .01, and *∗∗∗P* < .001.

**Table 2:** Oral examinations of TP in hemodialysis patients (n = 93).

Variable	
TP	93 (28.9)
Symmetry	
Symmetrical, n (%)	93 (100)
Left, n (%)	0 (0)
Right, n (%)	0 (0)
Location	
Incisor, n (%)	2 (2.2)
Premolar, n (%)	73 (78.5)
Molar, n (%)	18 (19.3)
Shape	
Flat, n (%)	52 (55.9)
Spindle, n (%)	21(22.6)
Nodular, n (%)	12 (12.9)
Lobular, n (%)	8 (8.6)
Size	
≥ 2cm, n (%)	46 (49.5)
< 2cm, n (%)	47 (50.5)

TP: torus palatinus.

**Table 3:** Comparison of the molar relationship and plaque index between hemodialysis patients with and without TP (n = 322).

Variable	All patients (n = 322)	Patients with TP (n = 93)	Patients without TP (n = 229)	*P* value
Molar relationship				0.198
None, n (%)	177 (55.0)	48 (51.6)	129 (56.3)	
Class I, n (%)	86 (26.7)	32 (34.4)	54 (23.6)	
Class II, n (%)	38 (11.8)	9 (9.7)	29 (12.7)	
Class III, n (%)	21 (6.5)	4 (4.3)	17 (7.4)	

Plaque index				0.336
No teeth, n (%)	13 (4.0)	1 (1.1)	12 (5.2)	
1, n (%)	8 (2.5)	1 (1.1)	7 (3.1)	
2, n (%)	44 (13.7)	11 (11.8)	33 (14.4)	
3, n (%)	160 (49.7)	52 (55.9)	108 (47.2)	
4, n (%)	79 (24.5)	24 (25.8)	55 (24.0)	
5, n (%)	18 (5.6)	4 (4.3)	14 (6.1)	

TP: torus palatinus.

**Table 4:** Predictors for TP in hemodialysis patients (n = 322).

Univariate analysis				Multivariate analysis		
Variable	OR	95% CI	*P* value	OR	95% CI	*P* value

Age	0.959	0.939 – 0.979	<0.001*∗∗∗*	0.960	0.939 – 0.982	<0.001*∗∗∗*

Female gender	2.155	1.339 – 3.469	0.002*∗∗*	2.305	1.374 – 3.867	0.002*∗∗*

Residual glomerular filtration rate	0.753	0.595 – 0.955	0.019*∗*	1.023	0.794 – 1.318	0.862

Phosphate	1.530	1.228 – 1.906	<0.001*∗∗∗*	1.411	1.110 – 1.794	0.005*∗∗*

Bicarbonate	0.833	0.750 – 0.926	0.001*∗∗*	0.887	0.791 – 0.994	0.040*∗*

CI: confidence interval; OR: odds ratio; TP: torus palatinus; *∗*P < .05, *∗∗*P < .01, and *∗∗∗*P < .001.

## Data Availability

The data used to support the findings of this study are available from the corresponding author upon request.

## References

[1] Komori T., Takato T. (1998). Time-related changes in a case of torus palatinus. *Journal of Oral and Maxillofacial Surgery*.

[2] Hsu C., Hsu C., Chang P. (2016). Oral tori in chronic peritoneal dialysis patients. *PLoS ONE*.

[3] Morrison M. D., Tamimi F. (2013). Oral tori are associated with local mechanical and systemic factors: A case-control study. *Journal of Oral and Maxillofacial Surgery*.

[4] Cortes A. R. G., Jin Z., Morrison M. D., Arita E. S., Song J., Tamimi F. (2014). Mandibular tori are associated with mechanical stress and mandibular shape. *Journal of Oral and Maxillofacial Surgery*.

[5] Padbury A. D., Tözüm T. F., Taba M. (2006). The Impact of primary hyperparathyroidism on the oral cavity. *The Journal of Clinical Endocrinology & Metabolism*.

[6] Rai S., Bhadada S. K., Rattan V., Bhansali A., Rao D. S., Shah V. (2012). Oro-mandibular manifestations of primary hyperparathyroidism. *Indian Journal of Dental Research*.

[7] Palla B., Burian E., Fliefel R., Otto S. (2018). Systematic review of oral manifestations related to hyperparathyroidism. *Clinical Oral Investigations*.

[8] Sisman Y., Gokce C., Sipahioglu M. (2012). Torus palatinus in end-stage renal disease patients receiving peritoneal dialysis: does renal osteodystrophy play a role?. *Journal of Dental Sciences*.

[9] Sisman Y., Ertas E. T., Gokce C., Akgunlu F. (2008). Prevalence of torus palatinus in cappadocia region population of Turkey. *European Journal of Dentistry*.

[10] Chao P.-J., Yang H.-Y., Huang W.-H. (2015). Oral tori in chronic hemodialysis patients. *BioMed Research International*.

[11] Gorsky M., Raviv M., Kfir E., Moskona D. (1996). Prevalence of torus palatinus in a population of young and adult Israelis. *Archives of Oral Biolog*.

[12] Jainkittivong A., Apinhasmit W., Swasdison S. (2007). Prevalence and clinical characteristics of oral tori in 1,520 Chulalongkorn University Dental School patients. *Surgical and Radiologic Anatomy*.

[13] Alkhadra T. (2017). Characteristic of malocclusion among Saudi special need group children. *Journal of Contemporary Dental Practice*.

[14] Turesky S., Gilmore N. D., Glickman I. (1970). Reduced plaque formation by the chloromethyl analogue of victamine C.. *Journal of Periodontology*.

[15] Bakathir A. A., Margasahayam M. V., Al-Ismaily M. (2008). Maxillary hyperplasia and hyperostosis cranialis. A rare manifestations of renal osteodystrophy in a patient with hyperparathyroidism secondary to chronic renal failure. *Saudi Medical Journal*.

[16] Lopes M. L. D. D. S., Albuquerque A. F. M., Germano A. R., Queiroz L. M. G., Miguel M. C. D. C., da Silveira É. J. D. (2015). Severe maxillofacial renal osteodystrophy in two patients with chronic kidney disease. *Journal of Oral and Maxillofacial Surgery*.

[17] Raubenheimer E. J., Noffke C. E., Mohamed A. (2015). Expansive jaw lesions in chronic kidney disease: Review of the literature and a report of two cases. *Oral Surgery, Oral Medicine, Oral Pathology, Oral Radiology, and Endodontology*.

[18] Hou Y.-C., Liu W.-C., Zheng C.-M., Zheng J.-Q., Yen T.-H., Lu K.-C. (2017). Role of vitamin D in uremic vascular calcification. *BioMed Research International*.

[19] Portillo M. R., Rodríguez-Ortiz M. E. (2017). Secondary hyperparthyroidism: pathogenesis, diagnosis, preventive and therapeutic strategies. *Reviews in Endocrine and Metabolic Disorders*.

[20] Harambat J., Kunzmann K., Azukaitis K. (2017). Metabolic acidosis is common and associates with disease progression in children with chronic kidney disease. *Kidney International*.

[21] Voiculet C. (2016). The role of oral sodium bicarbonate supplementation in maintaining acid-base balance and its influence on the cardiovascular system in chronic hemodialysis patients - results of a prospective study. *Journal of Medicine and Life*.

[22] Chiang M.-L., Hsieh Y.-J., Tseng Y.-L., Lin J.-R., Chiang C.-P. (2014). Oral mucosal lesions and developmental anomalies in dental patients of a teaching hospital in Northern Taiwan. *Journal of Dental Sciences*.

[23] Gorsky M., Bukai A., Shohat M. (1998). Genetic influence on the prevalence of torus palatinus. *American Journal of Medical Genetics*.

[24] Eggen S., Natvig B. (1986). Relationship between torus mandibularis and number of present teeth. *European Journal of Oral Sciences*.

[25] Reichart P. A., Neuhaus F., Sookasem M. (1988). Prevalence of torus palatinus and torus mandibularis in Germans and Thai. *Community Dentistry and Oral Epidemiology*.

[26] Haugen L. K. (1992). Palatine and mandibular tori: A morphologic study in the Current norwegian population. *Acta Odontologica Scandinavica*.

[27] Eggen S., Natvig B., Gåsemyr J. (1994). Variation in torus palatinus prevalence in Norway. *European Journal of Oral Sciences*.

[28] Bruce I., Ndanu T. A., Addo M. E. (2004). Epidemiological aspects of oral tori in a Ghanaian community. *International Dental Journal*.

[29] Yildiz E., Deniz M., Ceyhan O. (2005). Prevalence of torus palatinus in Turkish schoolchildren. *Surgical and Radiologic Anatomy*.

[30] Sawair F. A., Shayyab M. H., Al-Rababah M. A., Saku T. (2009). Prevalence and clinical characteristics of tori and jaw exostoses in a teaching hospital in Jordan. *Saudi Medical Journal*.

[31] Yoshinaka M., Ikebe K., Furuya-Yoshinaka M., Hazeyama T., Maeda Y. (2010). Prevalence of torus palatinus among a group of Japanese elderly. *Journal of Oral Rehabilitation*.

[32] Mishra N., Hiremath V., Husein A. (2011). Prevalence of torus palatinus and torus mandibularis among Malay population. *Journal of International Society of Preventive and Community Dentistry*.

[33] Sathya K., Kanneppady S. K., Arishiya T. (2012). Prevalence and clinical characteristics of oral tori among outpatients in Northern Malaysia. *Journal of Oral Biology and Craniofacial Research*.

[34] Simunkovic S. K. (2011). Prevalence of torus palatinus and torus mandibularis in the Split-Dalmatian County. *Collegium Antropologicum*.

[35] Auškalnis A., Bernhardt O., Putnienė E., Šidlauskas A., Andriuškevičiūtė I., Basevičienė N. (2015). Oral bony outgrowths: Prevalence and genetic factor influence. Study of twins. *Medicina*.

[36] Eggen S., Natvig B. (1994). Concurrence of torus mandibularis and torus palatinus. *European Journal of Oral Sciences*.

[37] Al-Bayaty H. F. (2001). An epidemiological study of tori among 667 dental outpatients in Trinidad & Tobago, West Indies. *International Dental Journal*.

[38] Romanos G. E., Sarmiento H. L., Yunker M., Malmstrom H. (2013). Prevalence of torus mandibularis in Rochester, New York, region.. *New York State Dental Journal*.

